# Supplementary material for: Ion implantation in nanodiamonds: size effect and energy dependence
Source: Sci Rep. 2018 Mar 23;8:5099. doi: 10.1038/s41598-018-23434-y (PMC5865192; doi:10.1038/s41598-018-23434-y)
Supplement: Supplementary file 1 — Supplementary information [file 41598_2018_23434_MOESM1_ESM.doc]

Ion implantation in nanodiamonds: size effect and energy dependence

A.A. Shiryaev^1,2*^, J.A. Hinks^3^, N.A. Marks^4^, G. Greaves^3^, F.J. Valencia^5,6^, S.E. Donnelly^3^, R.I. González^5,6^, M. Kiwi^5,6^, A.L. Trigub^7^, E.M. Bringa^8,9^, J. Fogg^4^, I.I. Vlasov^10, 11^

**Supplementary material**


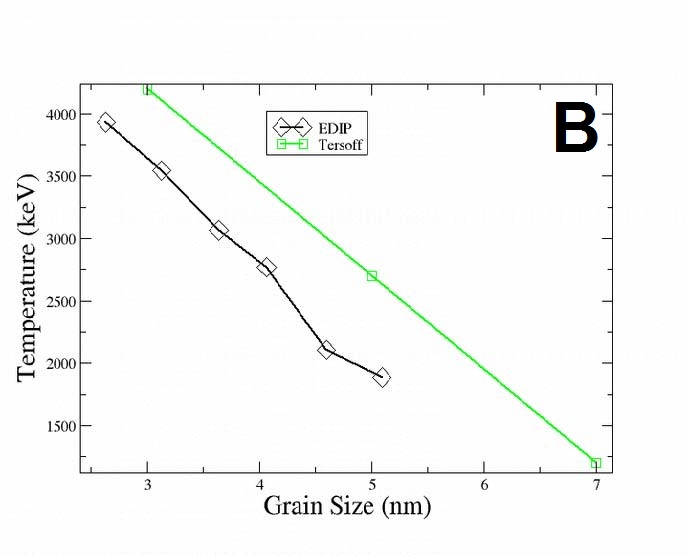

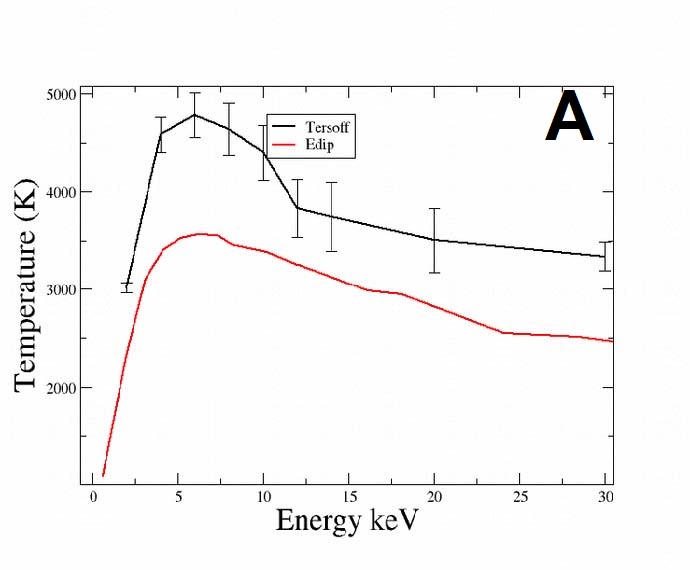
**Figure S1.** Comparison of results obtained using EDIP and LAMMPS (Tersoff) codes. Though absolute values of the temperature differ, the conclusions of the calculations are not influenced.

A - temperature of 3 nm nanodiamond grain after impact of Xe ions with different energies. B – temperature of ND grains of different sizes.


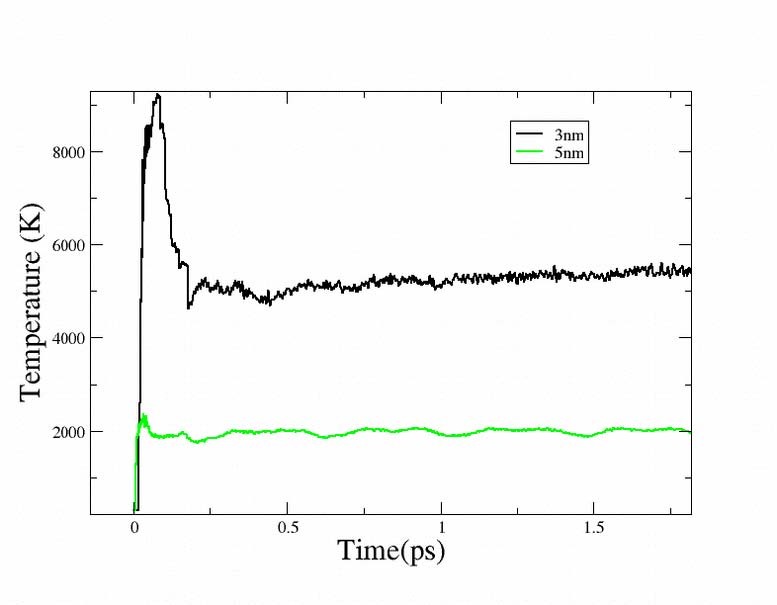


**Figure S2.** Evolution of nanodiamond temperature after impact of a Xe ion.

**Supplementary movies**

The movies show evolution of nanodiamond grains after the Xe ion impact. The ND size and Xe energy are indicated in filenames.
